# The Effect of L-Thyroxine Treatment on Hypothyroid Symptom Scores and Lipid Profile in Children with Subclinical Hypothyroidism

**DOI:** 10.4274/jcrpe.1594

**Published:** 2014-12-05

**Authors:** Gönül Çatlı, Ahmet Anık, Hale Ünver Tuhan, Ece Böber, Ayhan Abacı

**Affiliations:** 1 Dokuz Eylül University Faculty of Medicine, Department of Pediatric Endocrinology, İzmir, Turkey

**Keywords:** Subclinical hypothyroidism, children, dyslipidemia, LT4, hypothyroid symptom score

## Abstract

**Objective:** To evaluate i) the frequency of typical hypothyroidism symptoms in children with subclinical hypothyroidism (SH), ii) to evaluate the association of SH with lipoproteins and iii) to investigate possible improving effects of L-thyroxine (LT4) treatment on these findings.

**Methods:** Twenty-seven children with SH who had elevated thyroid-stimulating hormone (TSH: >4.94 µIU/L) but normal free T4 levels and healthy euthyroid children of similar age and sex were enrolled in the study. Anthropometric and laboratory (lipid profile and thyroid function tests) measurements were performed at diagnosis and six months after euthyroidism was achieved. All children were also subjected to a questionnaire on hypothyroid symptoms at diagnosis. The SH patients were subjected to the questionnaire also following treatment. Pre-treatment data were compared with those of controls and post-treatment measurements.

**Results:** Anthropometric and laboratory parameters of the groups were not statistically different except for higher TSH levels in the SH group. Serum lipoprotein levels and dyslipidemia frequency were similar between the groups. Compared to the controls, hypothyroidism symptom score was significantly higher in the SH group. Six months after euthyroidism was achieved, a significant reduction in the hypothyroid symptom score was obtained in the SH group. Except for significantly higher serum TSH values, no significant differences regarding demographic characteristics, symptom scores and lipid parameters were present between patients with Hashimoto’s thyroiditis and the remaining SH patients.

**Conclusion:** The results of this study showed that in children with SH i) the hypothyroidism symptom score was significantly higher than in euthyroid children, ii) LT4 treatment improved the hypothyroidism symptom score and iii) SH does not seem to be associated with dyslipidemia.

## INTRODUCTION

Subclinical hypothyroidism (SH) is defined by elevated serum thyroid-stimulating hormone (TSH) and normal serum free thyroxine (fT4) levels (1). It occurs in 5-15% of the general population and the reported prevalence of SH in childhood is 1.7-5.7% (2,3,4,5). Although patients with SH appear to be asymptomatic and suffering solely from a biochemical abnormality, it is well recognized that some individuals may present with typical symptoms and signs of hypothyroidism as well as metabolic (dyslipidemia, insulin resistance, etc.), neuromuscular, neurobehavioral alterations (5,6,7,8,9,10) and cardiac dysfunction (6,7,11,12,13,14,15). Many previous studies that were conducted in adults with SH have reported variable lipid profile results (16,17,18,19). Any beneficial effect of L-thyroxine (LT4) replacement on lipid profile in subjects with SH is also controversial (7,19,20).

Currently, limited data are available on lipid profile and symptom scores or on the possible effects of LT4 treatment in children with SH. With this study, we aimed i) to investigate whether SH is actually an asymptomatic laboratory diagnosis and ii) to explore the effects of SH and LT4 replacement on symptom scores and lipid profiles of children with SH.

## METHODS

This prospective study was conducted on patients attending the outpatient pediatric endocrinology clinic of our institution. Twenty-seven children with SH (SH group) and 24 euthyroid healthy children (control group) of similar age, pubertal status and sex were enrolled in the study. SH was diagnosed on the basis of elevated serum TSH levels and serum fT4 levels within the normal range. Hashimoto’s thyroiditis was diagnosed on the basis of the presence of either anti-thyroglobulin (anti-TG) or anti-thyroid peroxidase (anti­TPO) antibodies (or both) in the serum (21). Only patients with stable elevated TSH and normal fT4 levels in at least two different measurements 4-6 weeks apart were included in the study. Familial dyslipidemia, hepatic or renal dysfunction, diabetes mellitus, malignancy and obesity were excluded in both patients and the control group. In order to exclude any chronic disease, anemia or infection, we performed routine biochemical tests [kidney and liver function tests, Hitachi Modular Analytics (Roche, Tokyo, Japan)] and complete blood count [blood samples (K3-ethylenediaminetetraacetic acid) analyzed in an automated hematology analysis system (LH-780, Beckman Coulter, Brea, CA, USA) with the impedance method]. The results were normal in all of the study participants and none of them were taking any kind of medication for the last 3 months. Blood samples for determination of fT4, TSH, anti-TG and anti-TPO antibodies, total cholesterol (TC), high-density lipoprotein (HDL)-C, low-density lipoprotein (LDL)-C and triglycerides (TG) were obtained simultaneously in the morning after an overnight fast.

Height was measured using a Harpenden stadiometer with a sensitivity of 0.1 cm. Weight of the subjects was measured using a scale with a sensitivity of 0.1 kg (SECA, Hamburg, Germany), with all their clothing removed except undergarments. Body mass index (BMI) was calculated by dividing weight (kg) by height squared (m2).

Pubertal development was evaluated according to Tanner staging (22). A testicular volume of ≥4 mL in males and stage 2-5 of breast development in females were considered to be consistent with puberty.

Dyslipidemia was defined as lipid levels above the 95th percentile of healthy children (23).

Two experienced radiologists performed thyroid ultrasound examinations in all patients with SH to identify the etiological factors (thyroid parenchymal echogenicity, thyroid hypoplasia, etc.). The volume of each thyroid lobe was calculated with the formula: length (cm) x width (cm) x thickness (cm) x 0.52. Total thyroid volume was obtained by summation of the volumes of both lobes and was compared with the World Health Organization normative values (24). Patients with a serum TSH level higher than 4.94 μIU/mL and positive anti-TPO and anti-TG levels were diagnosed as autoimmune thyroiditis.

LT4 treatment was given to SH patients in a dose of 2 µg/kg/d and the dose was titrated every 4 weeks until a normal TSH level was maintained. Six months after euthyroid state was achieved, lipid measurements were repeated and the results were compared with the baseline values. The study protocol was approved by the ethics committee of the Turkish Ministry of Health and written informed consent was obtained from all participants and/or their parents.

**Serological Parameters**

Serum TSH and fT4 levels were measured by chemiluminescent microparticle immunoassay (CMIA) in Architec I2000SR analyser (Abbott Diagnostics Inc, Chicago, Illinois, USA). Anti-TG and anti-TPO antibodies levels were measured via a competitive radioimmunoassay (DYNOtest®, BRAHMS, Berlin, Germany). The coefficients of variation were 3.8% or less (intraassay) and 5% or less (interassay). The normal values in our laboratory are: fT4, 0.7-1.8 ng/dL; TSH, 0.35-4.94 mIU/L; anti-TG and anti-TPO less than 50

IU/mL. Fasting serum TG, TC and HDL-C concentrations were measured enzymatically using DP Modular Systems (Roche Diagnostic Corp., Indianapolis, IN, USA). LDL-C levels were calculated using the Friedewald formula when plasma TGs were <400 mg/dL.

**Hypothyroidism Symptom Score**

A questionnaire of 16 questions related to hypothyroid symptoms was administered to the study groups at inclusion. The questions were modified for children from a hypothyroid symptom questionnare comprising 19 questions for adults (25). A total symptom score was created by adding together the number of existing symptoms. At the end of the study, when the SH group became biochemically euthyroid for at least six months, the same questionnaire was repeated and a post-treatment symptom score was calculated.

**Statistical Analysis**

A commercially available statistical software package (SPSS 21.0 for Windows, Chicago, Ill., USA) was employed for all statistical analyses. The values are presented as median [Interquartile range (IQR)]. Statistical comparison between the groups was performed with the Mann-Whitney U test. Pre-treatment and post-treatment parameters were compared using Wilxocon test. Chi-square and McNemar’s tests were used for the comparison of categorical variables. The correlation between variables was evaluated using Spearman correlation analysis. A p-value of less than 0.05 was considered statistically significant.

## RESULTS

Twenty-seven children with SH (16 males, 15 prepubertal) with a median age of 10.0 years (6.9) and 24 euthyroid healthy children (10 males, 10 prepubertal) with a median age of 10.7 (5.0) years were enrolled in the study. The pre- and post-treatment symptom scores obtained by the questionnaire in the SH and control groups are given in Table 1. Total hypothyroid symptom score was significantly higher in the SH group than the control group (4 and 1, respectively, p<0.01). The answers given by the SH group differed from those of the controls regarding the following three questions on hypothyroid symptoms: i) Do you feel colder than other people? ii) Do you feel “pins and needles” in your hands or feet? iii) Do you have more shortness of breath than before? (p<0.05).

Table 2 shows the clinical and laboratory characteristics of the SH and control subjects. As expected, baseline serum TSH level was significantly higher in the SH group than controls (p<0.01) and serum fT4 level was not significantly different between the groups (p>0.05). Among the SH group, 3 patients had a TSH level above 10 mIU/mL and the rest of the group (n=24) had a TSH level between 4.94-10 mIU/mL. Serum TG, TC, LDL-C and HDL-C levels were not significantly different between the SH and control groups. Dyslipidemia frequency was similar between the study groups (p>0.05). Among the SH group, four subjects (14.8%) had hypertriglyceridemia, two (7.4%) had hypercholesterolemia, one (3.7%) had increased LDL-C levels and two (7.4%) had low serum HDL-C levels, while in the control group none of the participants had dyslipidemia.

**Figure 1:**
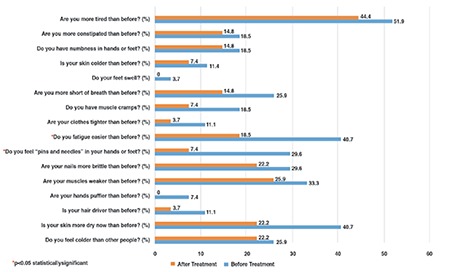
Percentage of subjects who answered yes to questions related to hypothyroidism in the subclinical hypothyroidism (SH) group before and after L-thyroxine (LT4) treatment

Euthyroid state was reached with a median (IQR) LT4 dose of 1.89 (0.6) µg/kg/d in a median (IQR) duration of 35 (30) days. Six months after euthyroid status was achieved, serum TSH levels decreased to the normal range and serum fT4 level showed a significant increase (pre-treatment, 1.1 (0.2) ng/mL; after-treatment, 1.3 (0.3) ng/mL; p<0.05). However, serum fT4 level remained within the normal range in all treated patients and no adverse effects or clinical signs of hyperthyroidism were observed during the entire treatment course. Besides, serum TG, TC, HDL-C and LDL-C levels were unchanged after LT4 treatment (Table 1). Two cases (7.4%) had hypertriglyceridemia, one (3.7%) had hypercholesterolemia and two (7.4%) had low serum HDL-C levels despite the achievement of a euthyroid state. After LT4 treatment, median (IQR) hypothyroidism symptom score significantly decreased [4 (2.0) and 1 (2.5), respectively, p<0.01], with a statistically significant decrease in scores of ‘pins and needles in hands or feet’ and ‘fatigue’ and a statistically non-significant decrease in score of ‘dry skin’ (Table 1, Figure 1). However, in the SH group, hypothyroidism symptom score was not significantly correlated with the TSH level (rho:-0.134, p=0.506).

The etiology of SH was Hashimoto’s thyroiditis in seven (26%) patients, thyroid hypoplasia in one (3.2%) patient and unknown (idiopathic) in 19 patients (70%). When patients with Hashimoto’s thyroiditis were compared with SH patients due to other etiologic factors, no significant differences regarding demographic characteristics, symptom score and lipid parameters were present (p>0.05), except for significantly higher serum TSH values in patients with Hashimoto’s thyroiditis [9.1 (8.1) and 6.9 (3.0), p<0.05] (Table 3).

## DISCUSSION

Thyroid hormones play an important role in growth, puberty and body metabolism. Along with typical clinical findings, short stature and retarded bone age are other findings of overt hypothyroidism (26). However, there are limited data regarding the effect of SH on growth and bone maturation. In a study by Cerbone et al (26) it was shown that persistent SH in children was not associated with alterations in growth, bone maturation, BMI and other complaints that could be ascribed to SH even after several years without therapeutic intervention. Similarly, in a study which prospectively evaluated 92 children with idiopathic SH, no patients showed any signs of hypothyroidism during follow-up and no changes in either height or BMI were observed despite the lack of LT4 treatment (27). On the other hand, in another study which investigated the effect of LT4 treatment in short children with SH, significant improvement in height and height velocity was reported (28). The results of our study showed that children with SH and euthyroid healthy children were not different in their anthropometric parameters. Since the median TSH level of the SH group was <10 mIU/L and the SH state was presumably of short duration, we cannot conclude that SH in childhood is not associated with anthropometrical deterioration.

In patients with overt hypothyroidism, there is an increase in serum TC, LDL-C, apolipoprotein B, lipoprotein (a) and TG levels. However, the effects of SH on serum lipid levels are not evident (18). The majority of data on SH and dyslipidemia was obtained from adult studies with conflicting results (19,29,30,31,32). SH has been detected in 1-11% of the cases with dyslipidemia (18). In a large study, Canaris et al (33) showed a positive correlation between serum TSH and lipid levels, suggesting SH as an intermediate state between euthyroidism and overt hypothyroidism in terms of lipid profile. In a recent study, a significant decrease in HDL-C level was reported in children with a TSH level >10 mIU/L, while a significant increase in TC and LDL-C levels was observed in adults with a TSH level >10 mIU/L and in contrast to these findings, no difference was observed in the lipid parameters of children and adults with a TSH level <10 mIU/L (16). Currently, there are limited data regarding the association between SH and lipid parameters in childhood and there are no data on the potential effect of LT4 replacement on lipid profile in children with SH. Studies conducted in adults with SH reported variable results about the changes in lipid parameters after LT4 therapy (7,16,17,18,19). In a meta-analysis which evaluated 148 studies investigating the effect of LT4 therapy on lipid parameters in SH, it was reported that hypercholesterolemia was 2-3 times more frequent in adults with SH as compared to euthyroid subjects and that LT4 therapy led to a 15 mg/dL decrease in TC levels of SH cases, independent of the baseline levels (19). Two randomized studies reported that TC levels showed no change with LT4 therapy (7,17). Asranna et al (34) found higher TC and LDL-C levels in adults with SH than in healthy controls and reported that LT4 therapy provided a significant decrease in TC, LDL-C and TG levels. In the present study, we observed no significant differences in lipoprotein levels between the SH and control groups and no change was documented after LT4 treatment. The lack of statistical significance could be explained by low number of subjects, a presumably short duration of SH period, or low number of patients with a TSH level above 10 mIU/mL in the SH group.

The classical signs of hypothyroidism are well-known and symptom scores have been developed to distinguish subjects to test for hypothyroidism (35,36). Although SH is proposed to be an asymptomatic biochemical diagnosis, in some cases, typical symptoms of hypothyroidism are reported (5). The Colorado Thyroid Disease Prevalence Study (33), which is the only large questionnaire-based study to investigate symptoms in patients with overt hypothyroidism and SH, reported a small but significant difference in symptoms between euthyroid and SH subjects (13.7% vs 12.1%). In that study, dry skin, poor memory, slow thinking, muscle weakness, tiredness, muscle cramps, feeling cold, deep and hoarse voice, puffy eyes and constipation were the main problems reported in patients with SH (33,35). Another randomized study showed that in adults with SH, the clinical symptom score showed a significant decrease after LT4 treatment (37). On the other hand, Cerbone et al (26) did not report any clinical finding of hypothyroidism in a group of adults with SH. To our knowledge, up to now, there is no study investigating the hypothyroid symptom score in childhood SH. The present study has shown that symptoms of hypothyroidism are significantly more frequent in patients with SH than in healthy children. Particularly for three questions [i) Do you feel colder than other people? ii) Do you feel “pins and needles” in your hands or feet? iii) Do you have more shortness of breath than before?], the SH group scored significantly higher than the healthy controls. Besides, LT4 replacement resulted in a significant decrease in total hypothyroidism symptom score, with a statistically significant decrease in scores of ‘pins and needles in hands or feet’ and ‘fatigue’. Although not statistically significant, a decrease in the score of ‘dry skin’ was also noted. These findings suggest that children and adolescents with SH are not totally asymptomatic and their symptoms should be questioned in more detail. In adults, it has been shown that with overt hypothyroidism, the number of symptoms correlated with the degree of hypothyroidism (35,36). Canaris et al (35) reported that the number of hypothyroid symptoms was directly related to the level of TSH. In our study, the hypothyroid symptom score was not significantly correlated with the TSH level (spearman rho:-0.134, p=0.506). This lack of association is most probably due to the fact that we only recruited children with SH and not with overt hypothyroidism.

Our study has some limitations that need to be acknowledged. First, the small number of study participants does not allow any conclusions that rule out a causal relationship between elevated TSH and lipid levels. Second, although there was no significant difference regarding anthropometric parameters between the SH and euthyroid healthy children, due to lack of an observation period prior to LT4 treatment, we are unable to comment on the effect of SH and of LT4 replacement on growth velocity. Thirdly, since a hypothyroid symptom score questionnaire developed particularly for children is not available, the questions in this study were modified by ourselves from the questionnaire form designed for adults.

In conclusion, although SH in childhood is known to be an asymptomatic process, the results of the present study have shown that in children with SH i) the hypothyroidism symptom score was significantly higher than in euthyroid healthy children, ii) LT4 treatment improved the hypothyroidism symptom score and iii) SH does not seem to be associated with dyslipidemia.

## Figures and Tables

**Table 1:**
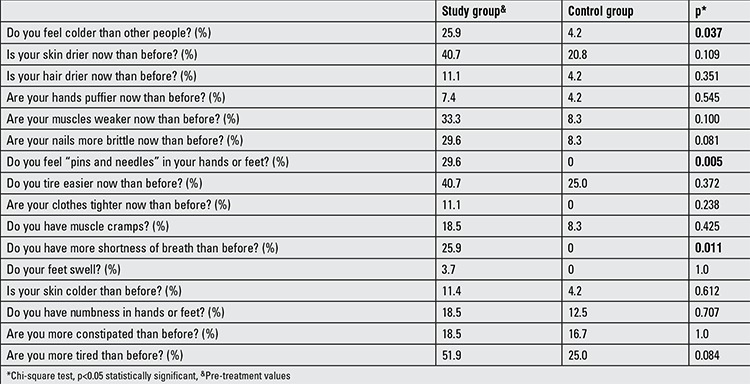
Hypothyroidism symptom scores in the study and control groups

**Table 2:**
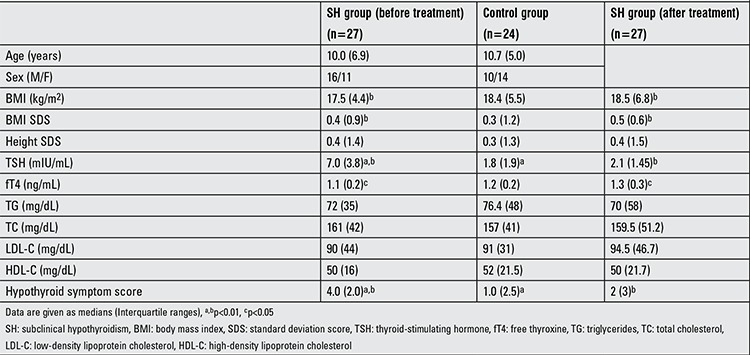
Clinical and laboratory characteristics of the SH and control groups

**Table 3:**
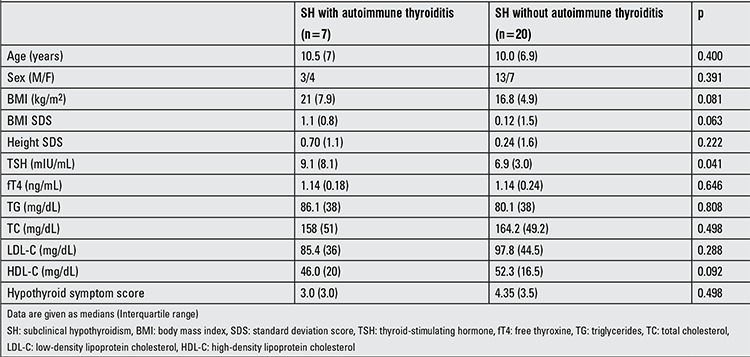
The clinical and laboratory characteristics of the SH subjects with and without autoimmune thyroiditis
